# Mesenchymal Stem Cells Pretreated with HGF and FGF4 Can Reduce Liver Fibrosis in Mice

**DOI:** 10.1155/2015/747245

**Published:** 2015-01-20

**Authors:** Sulaiman Shams, Sadia Mohsin, Ghazanfar Ali Nasir, Mohsin Khan, Shaheen N. Khan

**Affiliations:** ^1^National Centre of Excellence in Molecular Biology, 87 West Canal Bank Road, Thokar Niaz Baig, Lahore 53700, Pakistan; ^2^Stem Cells Regenerative Medicine Lab, Department of Biochemistry, Abdul Wali Khan University, Mardan, Khyber Pakhtunkhwa 23200, Pakistan

## Abstract

Stem cells have opened a new avenue to treat liver fibrosis. We investigated in vitro and in vivo the effect of cytokine (HGF and FGF4) pretreated MSCs in reduction of CCl_4_ liver injury. Mouse MSCs were pretreated with cytokines to improve their ability to reduce CCl_4_ injury. In vitro we gave CCl_4_ injury to mouse hepatocytes and cocultured it with untreated and cytokines pretreated MSCs. For in vivo study we labeled MSCs with PKH-26 and transplanted them into CCl_4_ injured mice by direct injection into liver. In vitro data showed that cytokines pretreated MSCs significantly reduce LDH level and apoptotic markers in CCl_4_ injured hepatocytes cocultured model. Furthermore the cytokines pretreated MSCs also improved cell viability and enhanced hepatic and antiapoptotic markers in injured hepatocytes cocultured model as compared to untreated MSCs. In vivo data in cytokines pretreated group demonstrated greater homing of MSCs in liver, restored glycogen storage, and significant reduction in collagen, alkaline phosphatase, and bilirubin levels. TUNEL assay and real time PCR also supported our hypothesis. Therefore, cytokines pretreated MSCs were shown to have a better therapeutic potential on reduction of liver injury. These results demonstrated the potential utility of this novel idea of cytokines pretreated MSCs for the treatment of liver fibrosis.

## 1. Introduction

Liver is a vital organ performing critical functions like urea synthesis, glycogen storage, hormone balance, and detoxification. The liver has an incredible regenerative ability but following chronic liver damage, it begins to fail and eventually develops fibrosis. Liver fibrosis is considered to be a process involving the progressive accumulation of collagen rich extracellular matrix (ECM). Scar formation due to wound-healing process is resolved in acute liver injury whereas in chronic liver injury repeated inflammation promotes the net deposition of fibrillar collagen [1]. Liver transplantation is the last available therapies at end stage fibrotic conditions. Lack of a donor, graft rejection, operative damage, and high cost are the factors which are making this procedure difficult.

Stem cell regenerative therapy is a promising approach in curing fibrosis. Bone marrow cells derived mesenchymal stem cells (MSCs) have a great potential in reducing fibrosis and improvement in the functions of lung, liver, heart, and brain when administered in these organs [2]. Adipose derived mesenchymal stem cells (MSCs) have recently been shown to promote liver repair and functions in liver damage [3, 4]. Transplantation of MSCs has also shown promising results in degradation of collagen in liver fibrosis [5].

Differentiation of MSCs into hepatocytes and intestinal cells was first identified through the detection of Y-chromosome-containing cells in samples from female recipients of BMCs from male donors [6, 7]. MSCs can differentiate into myocytes [8], chondrocytes, hepatocytes, osteoblasts, and adipocytes [9, 10]. Differentiation of MSCs towards hepatic lineage has been demonstrated by Wang et al. by only the induction of HGF [11]. Oyagi et al. transplanted bone marrow-derived mesenchymal stem cells (MSCs) treated with HGF and found reversal of liver injury in rats [12].

Some other reports have shown that transplantation of BMSCs could improve liver fibrosis, but their effects were insignificant [13]. While it has recently reported that bone marrow cells contribute to treat liver fibrosis in mice. Thus it is not clear until now which type(s) of stem cells has most effective in reduction of liver fibrosis [14].

We established MSCs culture and treated with HGF and FGF4 to increase their repair potential in liver fibrosis. HGF is a therapeutic agent increasing the essential capacity of tissues to regenerate. HGF plays a critical role in the development of liver cells, organ regeneration, wound healing, and MSCs differentiation toward hepatocytes. FGF4 is a prime candidate for autocrine signaling supporting self-renewal of stem cells. However the synergistic effect of HGF and FGF4 pretreated MSCs in liver regeneration is unknown. We employed CCl_4_-induced model of hepatocytes injury and analyze the ability of pretreated MSCs administration to improve fibrotic liver in vivo and injured hepatocytes in vitro. The present study was undertaken to examine the possible effects of HGF and FGF4 pretreated MSCs on CCl_4_ injured hepatocytes and liver fibrosis.

## 2. Materials and Methods

### 2.1. Culturing and Pretreatment of MSCs

Mesenchymal stem cells (MSCs) were isolated from femur and tibia of C57BL/6 mice according to the protocol described by [15] and were cultured in Iscove's modified Dulbecco's medium (IMDM, MP Biomedicals) with 20% FBS (Sigma) in the 25 mm culture flask. Medium was changed after every three days and MSCs were cultured to second passage. FACS analysis of the adherent MSCs using CD34, CD44, CD45, CD90, and CD105 antibodies was performed as described previously in our study [16] to confirm the presence of mesenchymal stem cells and to eliminate hematopoietic stem cells, after second passage cells division was blocked with serum-free medium for 24 hours. Then cells were plated at 1∗10^5^/well on collagen-coated 6-well plates in cytokines pretreated medium, which consist of LG-DMEM, 10% FBS, 20 ng/mL HGF, 10 ng/mL FGF4, 100 *μ*g/mL streptomycin, and 100 U/mL penicillin. Pretreated medium was changed every 3 days. MSCs cultured without cytokines medium were considered as control.

### 2.2. Morphological Analysis of Pretreated MSCs

Third passage MSCs were cultured in cytokines medium and cell morphology was analyzed at 0, 7, 15, 21, and 28 days using Olympus IX51 phase-contrast light microscope. MSCs cultured in cytokines-free medium were considered as negative control and culture of mouse hepatocytes were considered as positive control.

### 2.3. Analysis by RT-PCR

Total RNA was extracted from cytokines pretreated MSC using Trizol RNA isolation kit according to the manufacturer protocol (Invitrogen). The first strand cDNA was synthesized using 1 *μ*g of RNA and oligo-dT primers at 42°C for 60 minutes with a Reverse Transcription System (Fermentas kit). Polymerase chain reaction (PCR) was carried out on 1 *μ*L aliquots of cDNA and Taq polymerase using a standard PCR kit with specific primer pairs for mouse* Albumin*, Cytokeratin 8, Cytokeratin 18, and *β-actin*. The sequences of the primers were as follows (in Table 1). The PCR protocol consisted of 35 cycles at 94°C for 4 minutes, 56°C–58°C for 45 sec, and 72°C for 45 sec, followed by a final extension step at 72°C for 10 minutes. PCR products were size-fractionated on agarose gels and detected by ethidium bromide staining.

Gene expression levels of hepatic marker (*Albumin*, Cytokeratin 8) and apoptotic and antiapoptotic markers (*Bax*,* caspase-3*, and* Bcl-xl*) in injured hepatocytes/cytokines pretreated MSCs coculture model were also analyzed with RT-PCR, while analysis of gene expression in liver transplanted with cytokines pretreated MSCs was performed with quantitative real time PCR as described by our lab previously [16]. Comparative Ct method (ΔΔCt value) was used to determine the relative quantification of target genes, normalized to a reference gene (*β*-*actin*).

### 2.4. Immunostaining Assay

For immunostaining assay, cells were washed with PBS for three times and fixed in 4% paraformaldehyde for 30 min at room temperature and then blocked with 10% normal Donkey serum in PBS for 10 min to inhibit unspecific binding. The cells were washed with PBS and then incubated with various primary antibodies for 1-1/2 hr 37°C. The primary antibodies were used as follows: AFP, Goat polyclonal IgG (1 : 30; sc); Rb pAb to mouse serum* Albumin* (1 : 50; Abcam); CK8, mouse monoclonal IgG (1 : 30; sc); CK18, mouse monoclonal IgG (1 : 30, sc). The secondary antibodies were used according to the manufacturer's instructions: Texas Red-conjugated Donkey anti-Goat IgG (1 : 100; Jackson); FITC-conjugated Donkey anti-Rabbit IgG (1 : 100; Jackson); FITC-conjugated Donkey anti-mouse IgG (1 : 100; Jackson). The samples were incubated for 1 hr at 37°C and then washed with PBS and incubated with DAPI for 10 min at RT. At last cells were washed with PBS and mounted with vectasheet and examined under fluorescence microscope (Olympus IX51) and pictures were captured with Digital Camera DP-70.

### 2.5. Periodic Acid-Schiff (PAS) Stain for Glycogen

The PAS staining kit was purchased from Sigma Aldrich. Negative control (0 day) MSCs, positive control (mature hepatocytes) and cytokines pretreated for 15 days, and 28-day MSCs were fixed in 4% paraformaldehyde. For in vivo analysis 5 *μ*m thick microtome sections of liver were deparaffinized and incubated with periodic acid for 5 min at RT. Sections were washed with water and incubated with Schiff's reagent for 15 minutes and then with hematoxylin for 90 seconds and finally washed with tap water, mounted with cytoseal, and observed under microscope.

### 2.6. Hepatocytes Injury and Coculture Model

Hepatocytes were isolated from C57BL/6 mice by perfusing the liver with collagenase (type IV; Sigma Aldrich) according to the method of Seglen [17]. The viability of isolated hepatocytes was determined by the trypan blue exclusion test. Cells were cultured in RPMI medium supplemented with 100 U/mL penicillin, 100 *μ*g/mL streptomycin, 50 ng/mL EGF, and 10% FBS. Hepatocytes were plated at 1 × 10^5^/cm^2^ viable cells onto 6-well collagen-coated plate (BD) and incubated at 37°C in 5% CO_2_. The medium was replaced with serum-free medium after 2 hours of cell plating and CCl_4_ injury was induced after 24 hrs of plating. Hepatocytes cultured in absence of CCl_4_ for 6 hr were considered normal. The cultured medium was collected after 2 hr, 4 hr, and 6 hr of CCl_4_ injury and saved for LDH assay. MSCs cultured for 2 weeks in the presence or absence of HGF and FGF4 were detached from the plate and cocultured with injured hepatocytes. The untreated and cytokines pretreated MSCs were cocultured with injured hepatocytes for 48 hr in 10% FBS DMEM medium.

### 2.7. Cell Viability and Lactate Dehydrogenates (LDH) Assay

The cell viability of CCl_4_ injured and MSCs cocultured hepatocytes was calculated by the trypan blue negative cells by the total number of cells observed and then multiplied by 100. LDH activities in the supernatant obtained by centrifugation (50 g, 4 min) of hepatocytes medium were evaluated with LDH using a Sigma assay kit at 490 nm according to the manufacturing protocol.

### 2.8. Animals and Administration of CCl_4_

Mice used in the study were 7-to-8-week-old females. Mice were kept in sterile cages with free access to water and food. Ten mice were used for each experimental group. For CCl_4_-induced liver damage study, a dose of 1.0 *μ*L/g of body weight of CCl_4_ (1 : 1 in olive oil) was administered intra-peritoneally twice per week as described by [18].

### 2.9. Transplantation of Untreated and Cytokines Pretreated MSC

Untreated and day 15 cytokines pretreated MSCs were trypsinized at 37°C for 5 minutes with 0.25% trypsin and then labeled with PKH-26 Fluorescent Cell Linker Kit (Sigma Aldrich) according to the manufacturer's protocol and suspended in PBS. Animals were divided into four groups (*n* = 10) each. Group I mice were injected with saline, Group II contains CCl_4_ treated mice injected with saline and was considered as normal and control, respectively, while Groups III and IV consist of CCl_4_ treated mice transplanted with untreated and cytokines pretreated MSCs, respectively.

Approximately 1 × 10^6^ cells in 0.1 mL of suspension were transplanted into the left lateral and median lobes of female mice liver (*n* = 10). Three weeks after cell transplantation, the livers were removed and embedded in Tissue-Tek OCT compound (Sakura Torrance, CA, USA), and then sections were cut into 5 *μ*m thick slices with the help of cryostat (Microm) and checked for MSCs homing.

### 2.10. Histological Analysis and Measurement of Collagen

Liver tissues were fixed overnight in Paraformaldehyde Tissue Fixative, dehydrated through a series of ethanol treatments, and embedded in paraffin according to standard procedure. Sections were prepared and stained for collagen using Sirius Red [19]. In fibrotic area collagen was observed under Olympus BX-61 microscope and pictures were captured with Digital Camera DP-70 (Olympus, Japan). Quantitative analysis of collagen in Sirius Red-stained liver sections was performed by image J software to calculate the percentage of collagen in total liver tissue.

### 2.11. Enzyme Analyses

Blood samples were collected from hearts of each experimental mouse group and centrifuged at 8000 rpm for 15 min to isolate serum. Levels of alkaline phosphatase (ALP) and Bilirubin activity were measured in serum using the kit (Ecoline, Diagnostic System GmbH, Germany).

### 2.12. TUNEL Analysis

Apoptotic hepatocytes in the fibrotic liver were evaluated after 3 weeks of cell transplantation by TUNEL assay using cryosections using a TUNEL Apoptosis Detection Kit (Upstate Cell Signaling Solutions, Serological Company). TUNEL assay was performed in 5 *μ*m thick sections. More than 12 fields per liver lobe in each experimental group were examined under Olympus BX-61 microscope and images were taken with Digital Camera DP-70 (Olympus, Japan). In each section, four fields were selected for examination. The number of apoptotic hepatocytes was counted per high power field.

### 2.13. Statistical Analysis

All data are presented as mean ± SD. Significant differences were determined by using ANOVA in SPSS 16.0. *P* < 0.05 was considered statistically significant.

## 3. Results

### 3.1. Cytokines Pretreatment Induced Hepatocytes-Like Characteristic in MSCs

**Figure 1:**
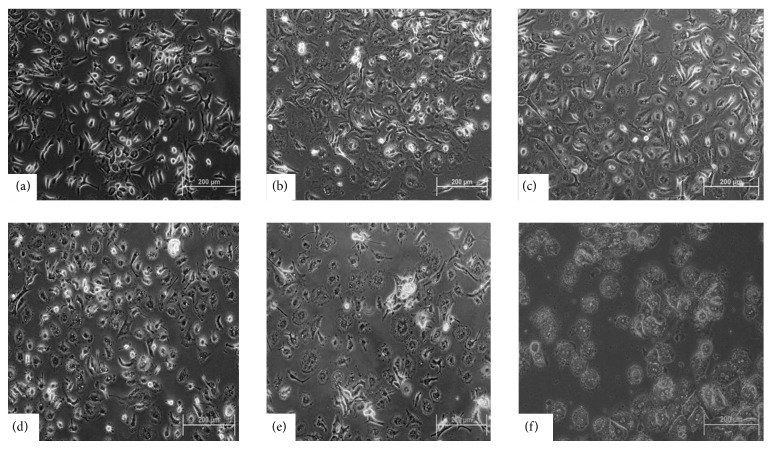
Morphological changes in cytokines pretreated MSCs: (a) MSCs as negative control; (b, c, d, and e) 7-, 15-, 21-, and 28-day cytokines pretreated MSCs; (f) mature hepatocytes as positive control (200x, scale bar ~200 *μ*m).

To determine the effect of cytokines pretreatment, morphology of differentiated MSCs was observed under phase-contrast microscope after every 7 d for 1 month. MSCs underwent a gradual morphological change from fibroblastic to hepatocyte-like polygonal shape from day 0 to day 28 of treatment (Figure 1).

**Figure 2:**
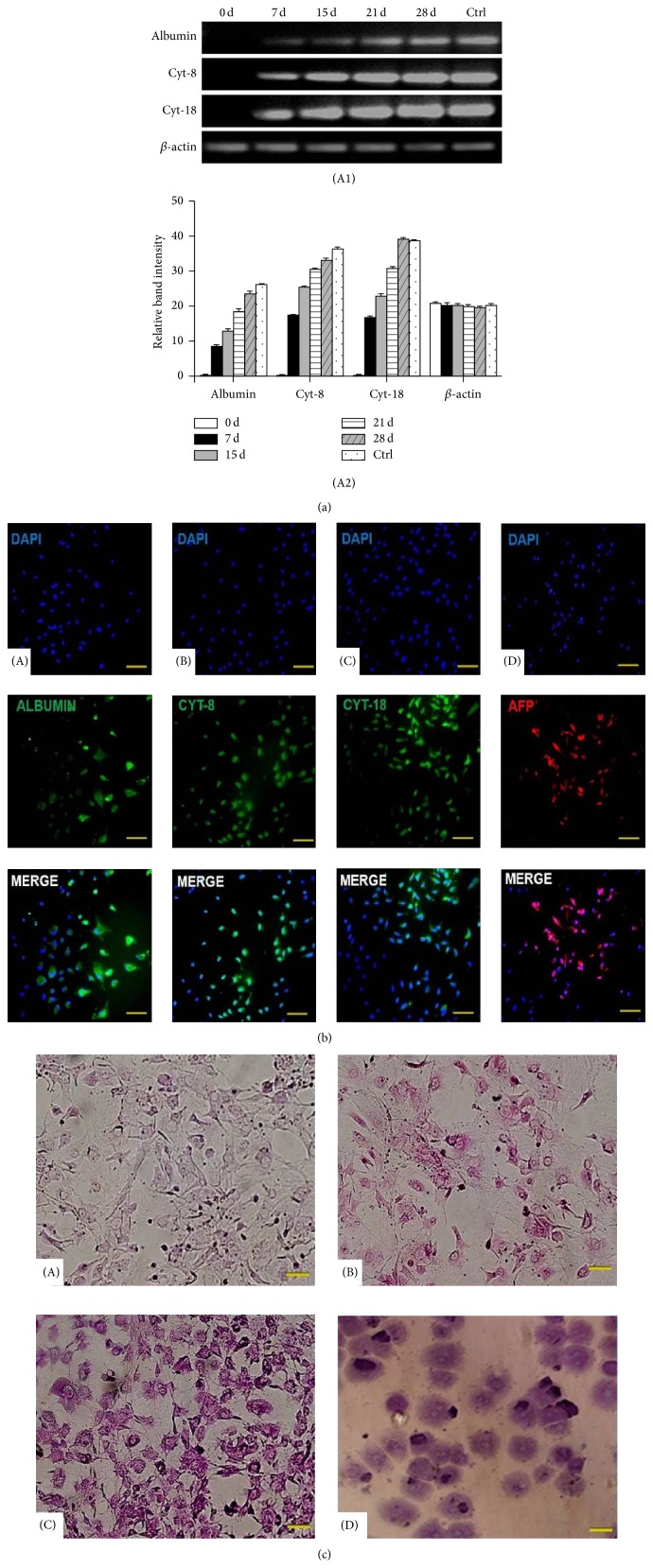
(a) Expression of hepatic markers: (A1) RT-PCR analysis of MSCs pretreated with cytokines (Lanes 1–5) 0, 7, 15, 21, and 28 days for the expression of* Albumin*,* Cyt-8, and Cyt-18*. (Lane 6), mature hepatocytes were used as positive control. (A2) Gel band quantification by Image J software. (b) Immunocytochemistry of cytokines pretreated MSCs. (A–D) Expression of hepatic markers at protein level for* Albumin*, Cytokeratin 8, Cytokeratin 18, and AFP on day 15, respectively. Nuclei were counterstained with DAPI (blue) (200x, scale bar ~100 *μ*m). (c) PAS staining after pretreatment for glycogen storage: (A, B, C) PAS staining of cytokines pretreated MSCs on days 0, 15, and 21. (D) Mature hepatocytes as positive control (200x, scale bar ~100 *μ*m).

To evaluate whether these morphological changes were associated with progressive differentiation towards hepatocyte-like cells, hepatic markers expression was analyzed at mRNA and protein levels. It was demonstrated by RT-PCR analysis in Figure 2(a) that the expression level of hepatic markers (ALB, CK-8, and CK-18) at RNA level increased steadily from day 7 to day 28 (Figures 2(a)(A1) and 2(a)(A2)). Further characterization of the cytokines pretreated differentiated MSCs by immunostaining demonstrated presence of hepatic markers like AFP (77 ± 2%), ALB (92 ± 2%),* Cyt-8* (88 ± 2.5%), and* Cyt-18* (84 ± 3%) (Figure 2(b)).

### 3.2. PAS Staining of Pretreated MSCs

For the assessment of hepatocytes-like functional activity of cytokines pretreated MSCs PAS staining was done. In Figure 2(c) PAS staining displayed that level of glycogen storage was increased in cytokines pretreated MSCs with the passage of differentiation time from d 0 to d 28 as compared to untreated MSCs.

### 3.3. Hepatocytes CCl_4_ Injury Model

**Figure 3:**
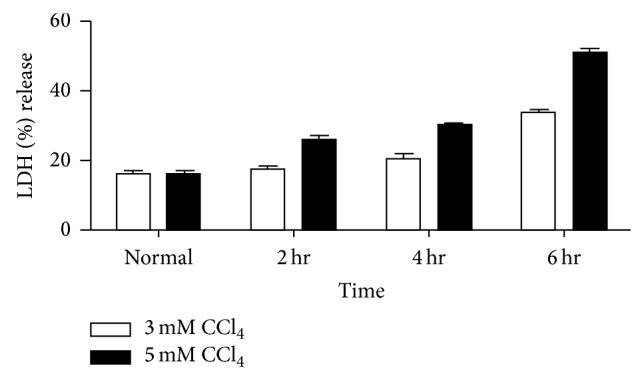
LDH assay of normal hepatocytes, CCl_4_ injured hepatocytes (3 mM, 5 mM) for 2 hrs, 4 hrs, and 6 hrs.

Hepatocytes injury model was established by exposing the cells to different concentrations (3 Mm and 5 Mm) of CCl_4_ for different intervals of time (2, 4, and 6 hours). LDH assay result showed that with the increasing of CCl_4_ concentration from 3 mM to 5 mM and time from 2 hr to 6 hr the injury level increases (Figure 3). RT-PCR analysis for the expression of hepatic (*Albumin* and* Cyt-8*), apoptotic (*Bax*,* caspase-3*), and antiapoptotic (*BCl-xl*) markers also confirmed the highest level of CCl_4_-induced hepatocytes injury level at 5 mM and 6 hr as described previously [20].

### 3.4. Cytoprotective Effects of MSCs on Injured Hepatocytes

**Figure 4:**
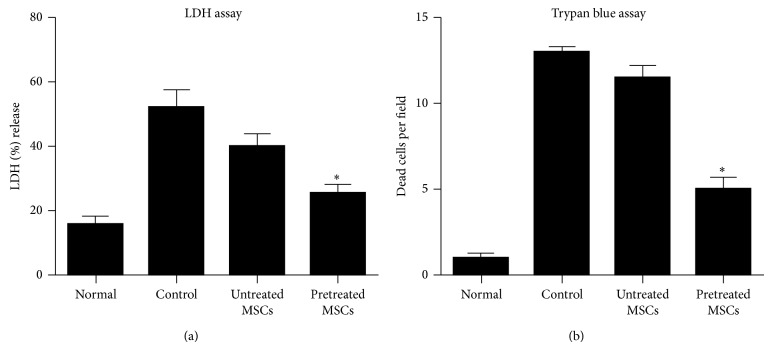
Effects of cytokines pretreated MSCs in injured hepatocytes cocultured model. (a) LDH release was estimated in the cell supernatant. (b) Cell viability was determined by counting damaged cells using trypan blue exclusion test. LDH release was reduced and the number of damaged cells was decreased in cytokines pretreated group as compared with control and untreated MSCs group. All values were expressed as mean + SEM. ^*^ *P* < 0.05 versus control and untreated MSCs group.

To determine the cytoprotective effect of cytokines pretreated MSCs for the reduction of CCl_4_ injury level; they were cocultured with injured hepatocytes.  Figure 4(a) demonstrated that CCl_4_ injury in hepatocytes was significantly reduced when cocultured with cytokines pretreated MSCs, as indicated by reduced LDH release as compared to control and untreated MSCs cocultured group. Similarly, cell viability assessed by trypan blue exclusion assay showed the lowest number of dead cells per field in cytokines pretreated MSCs group compared with the control and untreated MSCs cocultured group (Figure 4(b)).

### 3.5. Gene Expression Analysis of Hepatocytes MSCs Cocultured Model

**Figure 5:**
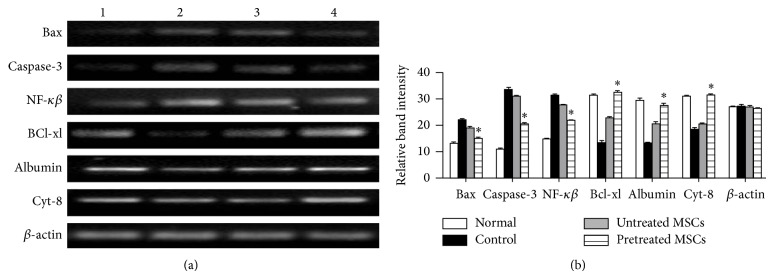
(a) Gene expression profile of MSCs/CCl_4_ injured hepatocytes coculture model: (Lane 1) normal hepatocytes, (Lane 2) CCl_4_ injured hepatocytes as control, and (Lanes 3 and 4) CCl_4_ injured hepatocytes cocultured with untreated and cytokines pretreated MSCs, respectively. (b) Quantification of gel band by image J software: ^*^ *P* < 0.05 versus control and untreated MSCs group.

The reduction of CCl_4_-induced hepatocytes injury by cytokines pretreated MSCs in cocultured model was also determined at RNA level with RT-PCR analysis.  Figure 5(a) demonstrated the cytokines pretreated MSCs compared to untreated MSCs significantly reduced the expression level of* Bax*,* caspase-3*, and* NF-κβ* markers and upregulated the expression level of* Albumin*,* Cyt-8*, and* BCl-xl* when cocultured with CCl_4_ injured hepatocytes. The gel bands were quantified with image J software (Figure 5(b)).

### 3.6. In Vivo Study

#### 3.6.1. Effects of MSCs Transplantation on Liver Fibrosis

**Figure 6:**
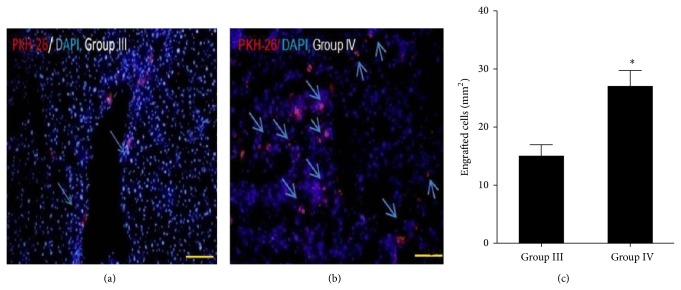
(a and b) Localization of untreated and cytokines pretreated MSCs in Group III and Group IV animals. (c) Quantification of engrafted cells in Groups III and IV. (100x, scale bar ~100 *μ*m). ^*^ *P* < 0.05 was considered to be significant.

**Figure 7:**
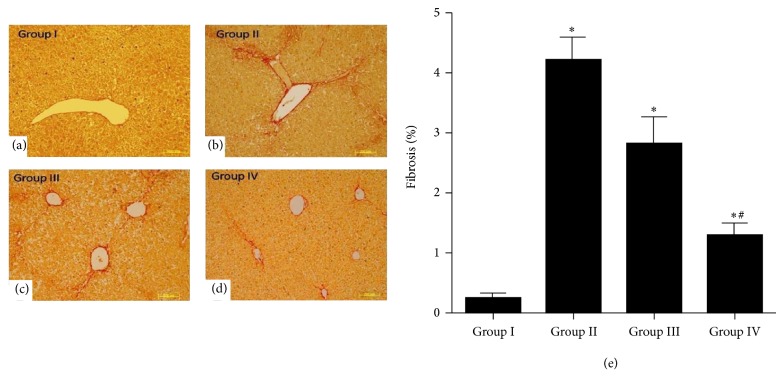
Sirius Red staining in liver section: (a–d) representing Groups I–IV; Sirius Red staining showed reduced collagen level in Group IV as compared to other groups (200x, scale bar ~100 *μ*m). (e) Bar graph represents percentage of fibrosis between the groups: ^*^ *P* was < 0.05 for Group I versus Groups II, III, and IV. ^#^ *P* < 0.05 for Group III versus Group IV.

To detect successful homing of MSCs in fibrotic liver, cytokines pretreated and untreated MSCs were labeled with PKH-26 fluorescence dye and were transplanted into left lateral lobe of Group III and Group IV animals, respectively (1 × 10^6^ cells/100 *μ*L/animal). After 3 weeks of transplantation mice were killed and transplanted MSCs were detected. Fluorescence microscopy showed that cytokine pretreated MSCs were significantly homed compared with untreated MSCs in fibrotic mice liver (Figures 6(a), 6(b), and 6(c)). We also analyzed liver histology using Sirius acid staining in mice transplanted with untreated and cytokines pretreated MSCs. Transplantation of cytokines pretreated MSCs significantly alleviated CCl_4_-induced fibrosis and collagen level compared with those in the control and untreated MSCs transplanted groups (Figures 7(a)–7(d) and 7(e)).

#### 3.6.2. Liver Function Improves after Cytokines Pretreated MSCs Transplantation

**Figure 8:**
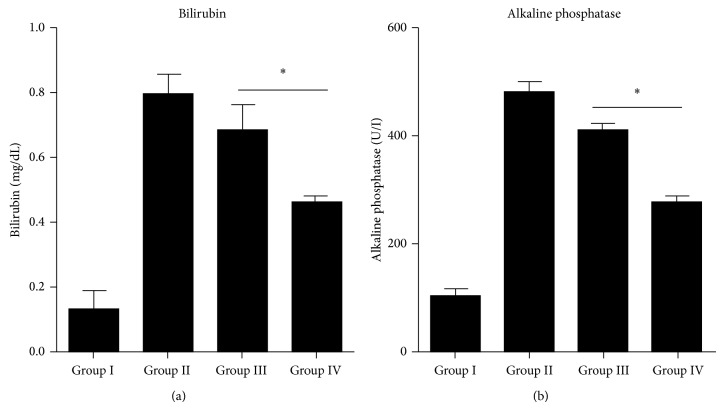
Functional analysis after MSCs transplantation: (a) bilirubin, (b) alkaline phosphatase; levels in Groups I, II, III, and IV after 3 weeks of transplantation. ^*^ *P* value < 0.05 was considered significant.

For assessment of liver functions after MSCs transplantation, serum ALP and Bilirubin levels across the four experimental groups were compared. Serum ALP and Bilirubin levels were significantly reduced in cytokines pretreated MSCs transplanted mice compared with control and untreated MSCs transplanted group mice (Figures 8(a) and 8(b)).

**Figure 9:**
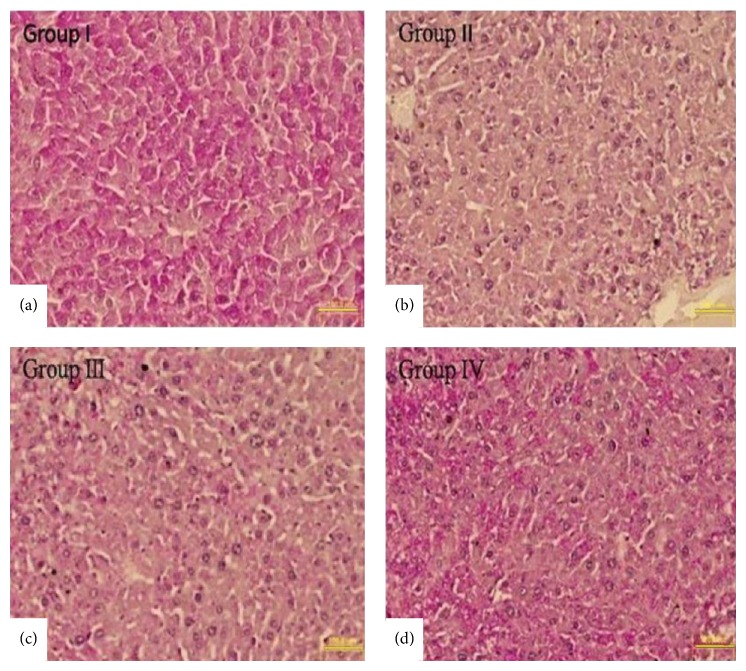
PAS staining for the storage of glycogen in liver sections of Group I to Group IV (a–d) (200x, scale bar ~100 *μ*m).

Glycogen storage is one of the vital functions of hepatocytes. PAS staining was performed to evaluate the glycogen storage level in all experimental groups.  Figure 9 demonstrated that in Group IV animals liver tissue the quantity of glycogen was the highest as compared to Group III and Group II.

#### 3.6.3. Effect of MSCs Transplantation on Apoptosis

**Figure 10:**
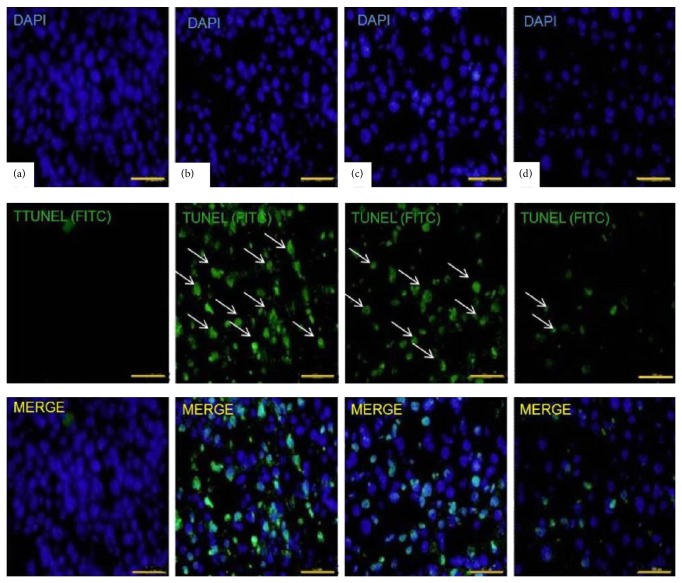
TUNEL apoptotic assay: assessment of apoptosis in experimental groups. (a–d) representing Groups I to IV: TUNEL-positive nuclei (green) in liver sections represent apoptotic cells. Nuclei were counterstained with DAPI (blue) (400x, scale bar ~100 *μ*m).

The number of TUNEL-positive cells in the fibrotic liver was significantly reduced in cytokines pretreated MSCs transplanted group (Figure 10(d)) when compared with the control group and untreated MSCs transplanted group (25 + 1.2 nuclei per field versus 45 + 1.8 and 37 + 2.9 nuclei per field), respectively (Figures 10(b) and 10(c)).

#### 3.6.4. Gene Expression Analysis after Cytokines Pretreated MSCs Transplantation

**Figure 11:**
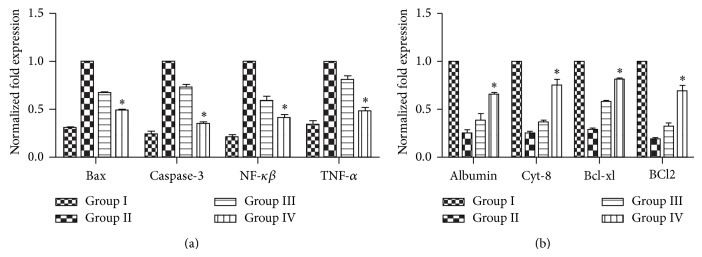
(a) Gene expression profiling after 3 weeks of transplantation: real time PCR analysis for apoptotic (*Bax, caspase-3, NF-κβ*, and*TNF-α*) genes between the experimental groups. The ^*^ *P* < 0.05 versus Group III and Group II. (b) Gene expression profiling after 3 weeks of transplantation: real time PCR analysis for hepatic (*Albumin, Cytokeratin 8*) and antiapoptotic (*Bcl-xl, Bcl*_*2*_) genes between the experimental groups. The ^*^ *P* < 0.05 versus Group III and Group II.

After 3 weeks of MSCs transplantation the expression levels of hepatic, apoptotic, and antiapoptotic markers were analyzed with quantitative real time PCR in all experimental groups.  Figure 11(a) demonstrated that transplanted cytokines pretreated MSCs significantly reduced the expression level of apoptotic (*Bax*,* caspase-3*, and* NF-κβ*) markers and upregulated the expression level of hepatic (*Albumin*,* Cyt-8*) and antiapoptotic (*BCl-xl*,* BCl*_2_) markers when compared to untreated MSCs transplanted group (Figure 11(b)).

## 4. Discussion

In the present report we revealed the efficacy of transplanting MSCs to treat liver failure in an experimental mouse model. Fluorescence activated cell sorting analysis showed that most of second passage MSCs were CD45, CD34 (−) and CD90, CD44, and CD105 (+) as we previously described [16]. Therefore, the hematopoietic stem cells involvement in reduction of liver fibrosis appears to be negligible.

Recent reports demonstrated that BMSCs transplantation attenuates liver fibrosis [18, 21]. On the other hand, [22] showed that bone marrow contributed functionally and significantly to liver fibrosis, and they identified bone marrow-derived activated hepatic stellate cells. These results seem conflicting.

Pretreatment of the MSCs culture with HGF and FGF4 before the transplantation improved the homing of MSCs in the recipient mice liver and caused clear therapeutic effects in CCl_4_ injury. In our present study, transplanted pretreated MSCs were successfully homed into mice liver and did not fade away after additional 3-week CCl_4_ treatment compared to untreated MSCs transplanted group as shown in Figures 6(a) and 6(b). PKH-26 labeled transplanted MSCs were still energetic and functional 3 weeks after CCl_4_ treatment, and the numbers of PKH-26 labeled cells remained almost similar to those at the end of CCl_4_ treatment (data not shown).

Although we did not evaluate the characteristics of homed MSCs in mice liver, it seemed that the transplanted pretreated MSCs were still immature and did not express sufficient cytochrome* P*-450 activity at the time of transplantation. In this regard, these pretreated MSCs enhanced synthesis of certain types of growth factors and cytokines and put forth a paracrine effect on local cellular dynamics, which supported the results of previous researchers [23, 24]. These bioactive substances might work in our experimental model, enhancing cell survival, and hence recover liver function.

In the present study, differentiated MSCs were checked at RNA level with RT-PCR using hepatic markers such as* Albumin*, Cytokeratin 8, and Cytokeratin 18 and at protein level with immunocytochemistry using AFP,* Albumin*, CK8, and CK18 antibodies. These markers were previously reported for hepatic genes expression studies [16, 25, 26]. It was showed in Figures 2(a)(A1) and 2(a)(A2) that the expression of these genes is increasing at RNA level in cytokines pretreated MSCs with the passage of time as they are going toward hepatic maturation.

In vitro results of PAS staining of cytokines pretreated MSCs also demonstrated the enhanced biological activity of cytokines pretreated differentiated MSCs. It has been previously reported that PAS staining is very useful technique to show glycogen storage ability of differentiated cells toward hepatic lineage [27, 28] and our results coincide with these reports. In vivo data of PAS staining demonstrated that high glycogen levels were restored in animals liver tissue transplanted with pretreated MSCs as compared to control and untreated group as shown in Figure 9. Significant reduction in the Bilirubin and ALP serum levels was also observed in experimental animals transplanted with pretreated MSCs compared to the control. We found that the transplantation of cytokines pretreated MSCs significantly reduced the serum Bilirubin and ALP levels and liver fibrosis in CCl_4_ injured mice.

Pretreatment of the MSCs culture with cytokines before BMSC transplantation appeared to be effective for the suppression of liver inflammation and fibrosis. PKH-26 staining showed that a significantly larger number of cytokines pretreated MSCs were homed in injured liver than untreated MSCs. Recently, Sakaida et al. [18] reported that the transplantation of freshly isolated bone marrow cells reduced the amount of established mouse liver fibrosis.

Our results coincide with the statement that bone marrow-derived hepatocytes-like cells have the best survival benefit over resident hepatocytes, and more repopulate the fibrotic liver of recipients [29]. Taking the above results together with the ones obtained in this study, we predict that bone marrow-derived cells will be useful for the treatment of liver fibrosis as well as the restoration of liver functions, such as Bilirubin and ALP production. In this paper, we have demonstrated the usefulness of cytokines pretreated MSCs for this therapeutic purpose.

Previously, we demonstrated that cytokines release from injured liver tissue promotes the transdifferentiation of MSCs into hepatic lineage cells and reduces liver fibrosis in mouse model when transplanted [16]. The present study confirmed that HGF and FGF4 are the key cytokines which are responsible for transdifferentiation of MSCs into hepatic lineage. Further these cytokines are also involved in enhancement of MSCs potential for the reduction of liver fibrosis.

In conclusion, HGF and FGF4 enhanced transdifferentiation of MSCs into hepatocytes-like cells both in vitro and in vivo. Moreover, the transplantation of HGF and FGF4 pretreated MSCs contributed to the improvement of liver function and in reduction of fibrosis in CCl_4_-induced mice liver fibrosis. MSCs can be simply and reproducibly harvested from patients by an easy and minimally insidious procedure. MSCs-based transplantation for patients with end stage liver fibrosis could therefore form a significant component of therapeutic strategies in this clinical field.

## Figures and Tables

**Table 1:** Primer sequences.

Gene	Primer sequence	Product size (bp)
Albumin (F)	GCTGTAGTGGATCCCTGGTG	196
Albumin (R)	GCTGTAGCCTTGGGCTTG	

Cyt-8 (F)	CTCACTAGCCCTGGCTTCAG	232
Cyt-8 (R)	ACAGCTGTCTCCCCGTGA	

Cyt-18 (F)	CACACTCACGGAGCTGAGAC	168
Cyt-18 (R)	GCCAGCTCTGACTCCAGATG	

Bax (F)	TGGAGATGAACTGGACAGCA	152
Bax (R)	CAAAGTAGAAGAGGGCAACCAC	

Caspase-3 (F)	TGTCATCTCGCTCTGGTACG	220
Caspase-3 (R)	AAATGACCCCTTCATCACCA	

TNF-*α* (F)	ACGGCATGGATCTCAAAGAC	162
TNF-*α* (R)	GGAGGTTGACTTTCTCCTGGTA	

NF-*κβ* (F)	GCACCTGTTCCAAAGAGCAC	200
NF-*κβ* (R)	GTGGAGTGAGACATGGACACAC	

Bcl-xl (F)	TTCGGGATGGAGTAAACTGG	150
Bcl-xl (R)	AAGGCTCTAGGTGGTCATTCAG	

Bcl2 (F)	GATGACTTCTCTCGTCGCTAC	182
Bcl2 (R)	ACGCTCTCCACACACATGAC	

*β*-actin (F)	GCTGTGTTGTCCCTGTATGC	106
*β*-actin (R)	GAGCGCGTAACCCTCATAGA	
